# Simultaneous tDCS-fMRI Identifies Resting State Networks Correlated with Visual Search Enhancement

**DOI:** 10.3389/fnhum.2016.00072

**Published:** 2016-03-07

**Authors:** Daniel E. Callan, Brian Falcone, Atsushi Wada, Raja Parasuraman

**Affiliations:** ^1^Center for Information and Neural Networks (CiNet), National Institute of Information and Communications Technology (NICT), Osaka UniversityOsaka, Japan; ^2^Multisensory Cognition and Computation Laboratory, Universal Communication Research Institute, National Institute of Information and Communications TechnologyKyoto, Japan; ^3^Center of Excellence in Neuroergonomics, Technology, and Cognition (CENTEC), George Mason UniversityFairfax, VA, USA

**Keywords:** fMRI, tDCS, resting state, functional connectivity, visual search, neuroergonomics

## Abstract

This study uses simultaneous transcranial direct current stimulation (tDCS) and functional MRI (fMRI) to investigate tDCS modulation of resting state activity and connectivity that underlies enhancement in behavioral performance. The experiment consisted of three sessions within the fMRI scanner in which participants conducted a visual search task: Session 1: Pre-training (no performance feedback), Session 2: Training (performance feedback given), Session 3: Post-training (no performance feedback). Resting state activity was recorded during the last 5 min of each session. During the 2nd session one group of participants underwent 1 mA tDCS stimulation and another underwent sham stimulation over the right posterior parietal cortex. Resting state spontaneous activity, as measured by fractional amplitude of low frequency fluctuations (fALFF), for session 2 showed significant differences between the tDCS stim and sham groups in the precuneus. Resting state functional connectivity from the precuneus to the substantia nigra, a subcortical dopaminergic region, was found to correlate with future improvement in visual search task performance for the stim over the sham group during active stimulation in session 2. The after-effect of stimulation on resting state functional connectivity was measured following a post-training experimental session (session 3). The left cerebellum Lobule VIIa Crus I showed performance related enhancement in resting state functional connectivity for the tDCS stim over the sham group. The ability to determine the relationship that the relative strength of resting state functional connectivity for an individual undergoing tDCS has on future enhancement in behavioral performance has wide ranging implications for neuroergonomic as well as therapeutic, and rehabilitative applications.

## Introduction

In recent years there has been an explosion of research investigating a method by which to augment human cognition by passing a low amplitude direct current (typically in the range of 0.5–2 mA) through a person’s head and enhancing human performance and abilities (Coffman et al., 2014). This technique is called transcranial direct current stimulation (tDCS). tDCS has been shown to enhance such abilities as attention and performance on vigilance, threat detection, and visual search tasks (Falcone et al., 2012; Parasuraman and Galster, 2013; Nelson et al., 2014); to enhance learning and performance on perceptual and cognitive tasks (Clark et al., 2012; Parasuraman and McKinley, 2014); and to improve motor and cognitive function in patients with brain damage, neuropsychiatric, and neurological diseases (Flöel, 2014; Kuo et al., 2014; O’Shea et al., 2014). The underlying neurological processes that allow for these enhancements in ability are under ongoing investigation. It has been shown that anodal DC stimulation decreases neural firing thresholds, and that glutamatergic modulation of long-term potentiation/depression may be involved with the enduring effects of tDCS (Liebetanz et al., 2002; Nitsche et al., 2003; Bikson et al., 2004; Coffman et al., 2014; Hunter et al., 2015). While one may expect these effects to be localized on the cortex near the stimulating electrode, functional MRI (fMRI) studies have also shown modulation in activity in distal brain regions suggesting possible network effects induced by tDCS (Clemens et al., 2014; Ellison et al., 2014; Weber et al., 2014).

It is our goal in this study to use simultaneous tDCS and fMRI to investigate the relationship between modulation in resting state activity as well as resting state functional connectivity of the brain correlated with improved performance as a result of stimulation. Studies have shown that resting state activity and connectivity in the brain can predict various characteristics such as attention (Kelley et al., 2008), learning (Baldassarre et al., 2012), memory (Hampson et al., 2006), language processing (Koyama et al., 2011), personality (Adelstein et al., 2011), and IQ (van der Heuvel et al., 2009; for review, see Stevens and Spreng, 2014). Previous studies using tDCS and fMRI have revealed, that as a result of stimulation, resting state networks can show wide spread changes in activity and connectivity in cortical and subcortical brain regions (Saiote et al., 2013; Clemens et al., 2014).

In our study, we investigate both resting state activity and performance related resting state connectivity in response to tDCS. A visual search task was employed before (pre-training), during (training), and after (post-training) tDCS stimulation to determine its facilitative effects on performance. Resting state fMRI was recorded toward the end of each session after completing the visual search task. We placed the stimulating electrode over the posterior parietal cortex as it has been found in previous tDCS studies to modulate visual search performance (Bolognini et al., 2010; Ellison et al., 2014). We used the fractional amplitude of low frequency fluctuations (fALFF) in the BOLD signal, which has been found to be associated with spontaneous neural activity (Biswal et al., 1995; Zou et al., 2008; Song et al., 2011), as a measure of resting state activity. By comparing fALFF across tDCS stimulation and sham groups we intend to show brain regions in which the spontaneous neural activity is being modulated. Unlike most previous neuroimaging studies, we applied tDCS and fMRI concurrently in order to observe the active effects of tDCS on resting state activity rather than just the after-effects that exist following the cessation of tDCS. Brain regions determined to show tDCS induced activity are then used as seed regions for a functional connectivity analysis (Song et al., 2011). It is hypothesized that resting state functional connectivity related to improvement in behavioral performance on the visual search task will be found to exist for these seed regions for the tDCS group to a greater extent than for the sham group.

Our study addresses many of the future directions concerning the investigation of tDCS on resting state activity and connectivity proposed by Clemens et al. (2014). Specifically, we applied tDCS and fMRI concurrently to investigate the immediate active effects on resting state activity and connectivity. The after-effects of tDCS on resting state functional connectivity were also investigated following a post-training session. In addition, as proposed by Clemens et al. (2014), our study includes the use of sham stimulation. By comparing between tDCS stim and a sham group (unlike other studies that look at tDCS stim vs. pre-stim), our study is able to investigate behaviorally related enhancement in resting state functional connectivity that differs between the two groups that can be attributed to modulation by tDCS rather than changes in resting state connectivity that normally occur as a result of task training.

## Materials and Methods

### Participants

There were 28 participants that took part in this study. All of the participants (14 males, 14 females) were Japanese right-handed adults ranging from 18 to 25 years (mean = 20.7) of age from Osaka University. The participants were pseudo-randomly assigned to the tDCS stim and sham groups such that there were seven females and seven males in each group. All participants were screened for exclusion if there was a history of head injury, history of mental, neurological, alcohol or drug abuse disorders, or using medication that affects central nervous system function. The participants gave written and informed consent to take part in this experiment. The experimental procedures were approved by the National Institute of Information and Communications Technology (NICT) Human Subject Review Committee were carried out in accordance with the principles expressed in the WMA Declaration of Helsinki. Originally there were 18 tDCS stim and 17 sham participants. From the tDCS stim group two participants were excluded because of pressure pain caused by the tight fit of the headphones within the head coil and two participants were excluded because task performance was below chance on session three even after completing the training session. From the sham group one participants was excluded because of pressure pain caused by the tight fit of the headphones within the head coil and two participants were excluded because task performance was below chance on session three even after completing the training session.

### Procedure

The experiment consisted of three sessions within the fMRI scanner. During the first part of scanning the participants conducted a visual search task. During the last 4.5 min of fMRI scanning, for each session, resting state activity was acquired. In this study, we will focus only on the resting state fMRI data from these sessions.

The visual search task was based on a search and rescue mission that required participants to locate a red pickup truck located in the search area amongst buildings and other similar looking distractor vehicles. In each trial there were five non-moving vehicles distributed throughout the search area, one of which could be the red truck. There were a total of 60 trials in each session and the target red truck was randomly present on half of the trials. The task was designed so that as the unmanned aerial vehicle UAV loitered in a circle around the search area, all vehicles would remain in constant view despite a continually changing view angle. Each trial lasted 10 s where the participants searched the area looking for the target and were required to make a button press indicating whether the search area contained a target or not.

The three experimental sessions consisted of the following: Session 1: Pre-training session that did not provide performance feedback. Session 2: Training session in which tDCS stimulation or sham stimulation was delivered. In the training session immediate reinforcement error-feedback (“ding” sound correct, “buzz” sound incorrect) after each response. Additionally, for target present trials only, a transparent white sphere would appear over the target at the end of the 10 s trial identifying the target location. This type of auditory reinforcement feedback will allow subjects to know immediately whether the features they were attending to are incorrect, in the case of a false alarm or a miss, or correct, in the case of a correct rejection or a hit. This information together with the visual feedback of the position of the target at the end of the trial when it was present will allow for learning of the relevant features and improve performance. Session 3: Post-training session with no feedback. The total time of the visual search task for session 1 and 3 was approximately 15 min and session 2 was approximately 16 min. After each experimental session resting state activity was recorded for 4.5 min. For session 2 tDCS stimulation was given concurrently with fMRI scanning. The task for the participants during collection of the resting state data was to visually fixate on a white cross mark presented in the center of the display against a black background. Participants were instructed to fixate on the cross on the screen, and to relax without falling asleep.

### Transcranial Direct Current Stimulation

**Figure 1:**
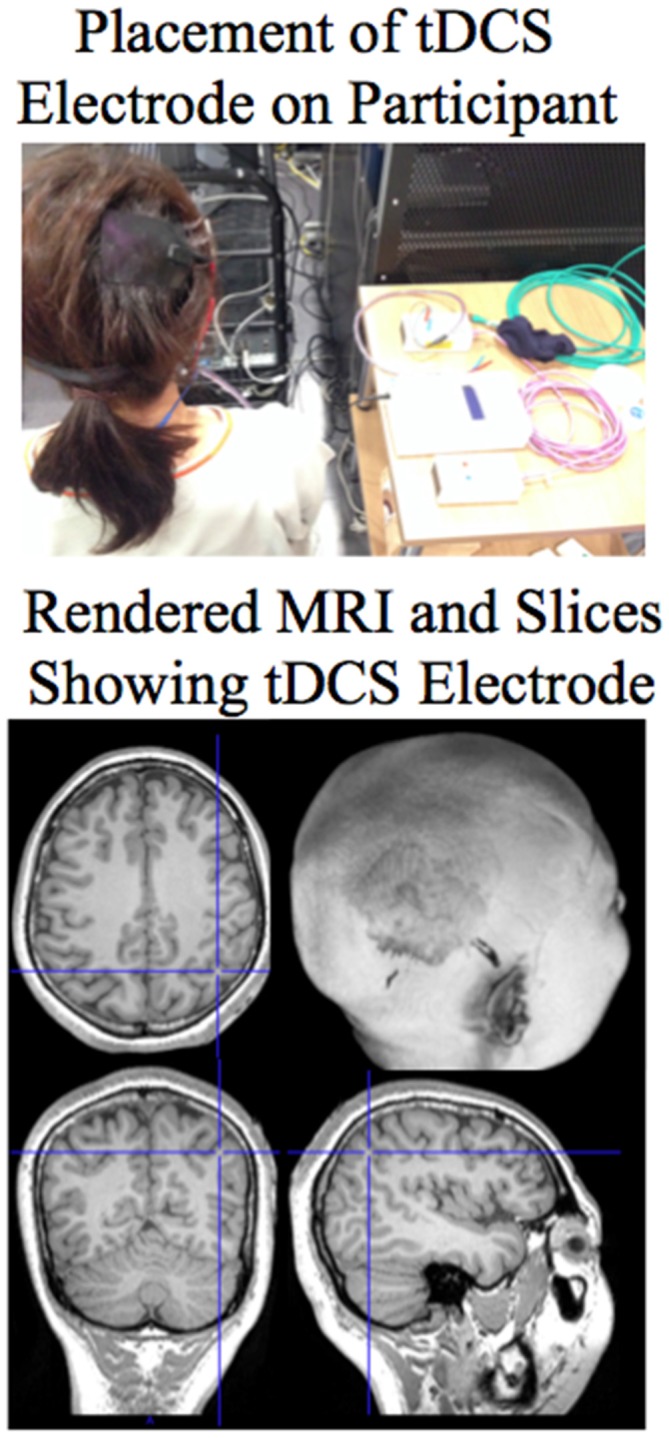
**Top**: Picture showing the placement of the anodal tDCS electrode on the right posterior parietal cortex of the participant. **Bottom**: The placement of the tDCS electrode can be seen in the rendered MRI of the participant. Sections are shown through the brain at the site of the electrode. For the MRI sections the right side of the image is the right side of the brain.

TDCS was delivered during the training session (session 2) using the MRI compatible NeuroConn DC-Stimulator MR. Two rectangular-shaped (5.3 × 7.2 cm) MRI compatible conductive rubber electrodes were placed on the participant before entering the MRI scanner (see Figure 1 for picture of placement of electrode on the head of a participant and a rendered MRI showing the tDCS electrode on the head). The anodal electrode was placed over the right posterior parietal cortex. It was placed over where the P4 electrode is located according to the 10–20 International EEG System. The electrode was held in place by the conductive paste (Ten20 conductive paste gel, Waver and Company) as well as a padded headband. The cathodal electrode was placed over the contralateral left side trapezius muscle on the back of the shoulder.

Participants in the stim group received 1 mA current for a total of 30 min (1 mA was the highest level possible within the MRI scanner with our version of the NeuroConn DC-Stimulator MR). Stimulation was started 5 min before the task in order to ensure that the full modulatory effect of tDCS was active during task performance. Current was ramped up over the initial 10 s and ramped down the last 10 s of stimulation. The participants in the sham group also received 1 mA current but only for 30 s and then the unit was turned off. This procedure helps to conceal from the participant which group (stim or sham) they belong to as both groups feel the onset of the stimulation. In addition group membership of the participant was not known by the experimenter giving the instructions.

### fMRI Data Collection and Analysis

fMRI scanning of resting state activity was acquired for 4.5 min at the end of each session (TR = 2 s; 30 interleaved slices covering the brain and cerebellum, 3 mm × 3 mm × 4 mm voxels; Siemens 3T Trio Scanner; 32 Channel head coil). Preprocessing of fMRI data was conducted using SPM8 (Wellcome Department of Cognitive Neurology, UCL) and included realignment and unwarping, normalization to the template EPI image (2 mm × 2 mm × 2 mm), and smoothing (8 mm × 8 mm × 8 mm). EPI template based normalization was used because the source image upon which the normalization parameters are determined is in the same space as the scans to be normalized. This has advantages in that it avoids additional steps of coregistration to/or from EPI space (resulting in image distortion) that are required when using an anatomical T1 or T2 image to determine the normalization parameters. Because whole brain EPI was acquired the difficulties associated with mapping partial EPI volumes to the template image are avoided.

The REST (Song et al., 2011) Toolkit was used to conduct the resting state spontaneous activity (fALFF) and the functional connectivity analyses. The realignment parameters were used as covariates of non interest and regressed out of the preprocessed EPI data to extract potential confounds related to head movement while scanning. The linear trend was then removed from the data. The parameters for the fALFF analysis included a low frequency fluctuation band of 0.01–0.08 Hz (Biswal et al., 1995) compared to the entire frequency range (0–0.25 Hz). The fALFF results were normalized by dividing by the mean fALFF values within the whole brain mask to be used for second level random effects analyses. The functional connectivity analysis was carried out over the preprocessed covariates removed detrended and filtered (0.01–0.08 Hz) data. Three separate functional connectivity analyses were conducted. The seed regions of interest (ROI) were determined from the results of the fALFF analysis (see “Results” Section). The ROI included the precuneus (MNI 6, −46, 60). A Spherical region with a radius of 8 mm at the given coordinate was used as the seed for the resting state functional connectivity analyses conducted separately for each session. The Pearson linear correlation was used to determine the functional connectivity between the mean of the voxels within the seed ROI and the rest of the voxels in the brain according to the defaults in the REST toolbox (Song et al., 2011). The Fisher’s z transform was used to normalize the correlation coefficients to be used for second level random effects analyses. SPM8 was used to conduct the random effects analyses. Correction for multiple comparisons (*p* < 0.05) across the entire brain was carried out using Monte-Carlo simulation of the brain volume to define a voxel contiguity threshold at an uncorrected significance level of *p* < 0.005 (Slotnick et al., 2003; Ellison et al., 2014). Using 10000 Monte-Carlo simulations a cluster extent greater than 154 voxels thresholded at *p* < 0.005 uncorrected, is necessary to correct for multiple comparisons across the whole brain at a threshold *p* < 0.05. Activated brain regions were identified using the SPM Anatomy Toolbox v1.8 (Eichkoff et al., 2005) as well as Talairach Client after transforming from the MNI to the Talairach coordinate system using mni2tal function in Matlab. The substantia nigra, red nucleus, and subthalamic nuclei were identified using the regions specified in Keuken and Forstmann (2015).

## Results

### Behavioral Results

The behavioral results in terms of percent correct on the visual search task for the tDCS stim and sham groups are as follows: there was a significant enhancement in performance post- relative to pre-training (ANOVA *F*_(2,52)_ = 12.47, *p* < 0.05). The enhancement was statistically significant (*t* = 4.05; *p* < 0.05) for the stim group (pre-training mean = 64.26%; SE = 2.64; post-training mean = 72.06%; SE = 2.36) and was statistically significant (*t* = 3.15; *p* < 0.05) for the sham group (pre-training mean = 64.98%; SE = 2.47; post-training mean = 73.02%; SE = 1.99). There was no significant difference (assessed at *p* < 0.05) between stim and sham groups for either pre- (*T* = −0.21) or post-training (*T* = −0.33) sessions. The interaction between stim and sham groups and pre- and post-training session was not significant (*F*_(2,52)_ = 0.3; *p* > 0.05). Additionally there was no significant difference (*T* = −0.83, *p* > 0.05) between stim (mean = 64.86%; SE = 2.7) and sham (mean = 67.94%; SE = 2.74) groups for the training session 2.

### Brain Imaging Results

#### Resting State Activity: fALFF Analysis

**Figure 2:**
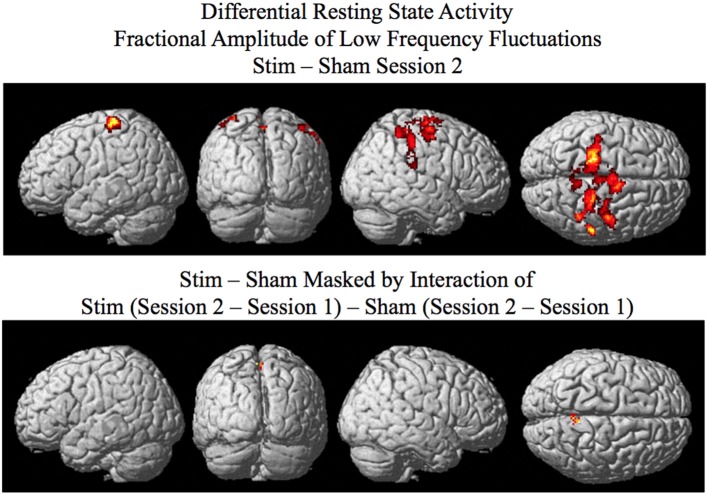
**Results of the SPM random effects analysis rendered on the surface of the brain.** Top: Differential resting state activity as measured by fractional amplitude of low frequency fluctuations (fALFF) for the stim–sham groups for session 2 corrected for multiple comparisons at the cluster level (*p* <0.05) using Monte-Carlo simulation (corrected cluster extent threshold greater than 154 contiguous voxels over uncorrected significance threshold of *p* < 0.005). Bottom: Activity in the session 2 stim–sham analysis above that is additionally masked by the interaction of Stim (Session 2 – Session 1) – Sham (Session 2 – Session 1) corrected (*p* < 0.05) for multiple comparisons at the cluster level.

The results of the fALFF analysis are given in Figure 2 and Table 1. Using a random effects between groups *t*-test, statistically significant (*p* < 0.05 corrected for multiple comparisons, see “Materials and Methods” Section) differences in fALFF between the stim and sham groups for session 2 were found to be located in three clusters of activity: Cluster 1 is located around the right superior parietal spreading into the left parietal cortex as well as the neighboring regions of the precuneus, post central gyrus, pre-central gyrus, and supplementary motor area (It should be noted that since the analysis is corrected for multiple comparisons at the cluster level we cannot definitively know which of these regions making up the cluster are activated, only that some of them are); Cluster 2 is located in the right inferior parietal lobule including the temporal parietal junction; Cluster 3 is located in the premotor cortex BA6 (see Figure 2, top and Table 1, top). Brain regions statistically significant (*p* < 0.05 corrected) for the stim–sham contrast masked by the interaction (random effects ANOVA) of Stim (Session 2 – Session 1) – Sham (Session 2 – Session 1) with a corrected threshold of *p* < 0.05, consisted of the right precuneus (see Figure 2, bottom and Table 1, bottom). Masking by the interaction controls against differences in fALFF that may exist between the tDCS stim and sham groups prior to training. In addition the masking also allows for a more focal site that is likely modulated by the tDCS, which can be used as a seed for the resting state functional connectivity analyses. No statistically significant differences in fALFF were found for session 2 for the sham greater than stim contrast when correcting for multiple comparisons.

**Table 1:** **Differential resting state activity between stim and sham groups determined by fractional amplitude of low frequency fluctuations**.

Brain region	MNI coordinates *x*, *y*, *z*	*T* (*df* = 26)	*Z*-score	Cluster size
**fALFF (Stim–Sham) session 2**
Precuneus BA7	6, −46, 60	3.76	3.33	1902
SPL BA40	40, −44, 54	3.99	3.76	
Postcentral Gyrus BA3	24, −32, 60	4.48	3.82	
Postcentral Gyrus BA1, 6	−22, −32, 54	4.49	4.49	
SMA BA6	6, 2, 66	3.89	3.42	
IPL BA40	58, −30, 44	5.17	4.25	192
TPJ BA40	50, −30, 22	3.88	3.42	
Precentral Gyrus BA4, 6	46, −14, 62	4.29	3.70	291
Premotor Cortex BA6	26, −16, 74	3.60	3.21
**fALFF (Stim–Sham) session 2 masked by interaction**
Precuneus BA7	6, −46, 60	3.76	3.33	

*Top: Brain regions showing significant differential activity for the stim–sham comparison for session 2 corrected for multiple comparisons at the cluster level (p < 0.05) using Monte-Carlo simulation (corrected cluster extent threshold greater than 154 contiguous voxels over uncorrected significance threshold of p < 0.005). Bottom: Regions that are also significant when masking the results by the interaction of Stim (Session 2 – Session 1) – Sham (Session 2 – Session 1) corrected for multiple comparisons at the cluster level (p < 0.05). BA, Brodmann area; IPL, Inferior Parietal Lobule; SPL, Superior Parietal Lobule; SMA, Supplementary Motor Area; TPJ, Temporal Parietal Junction. Negative “x” MNI coordinates denote left hemisphere and positive “x” values denote right hemisphere activity*.

#### Resting State Connectivity: Functional Connectivity Analysis

Resting state functional connectivity analyses for each of the three sessions, using post relative to pre behavioral performance as a covariate of interest, were conducted using the precuneus (significant for the fALFF analyses; see Figure 1, bottom and Table 1, bottom) as a seed. In order to determine differences between the tDCS stim and sham groups in resting state functional connectivity related to enhanced behavioral performance, a random-effects between groups *t*-test using post-minus pre-training behavioral performance as a covariate of interest was conducted. Enhancement in behavioral performance was defined as the percent correct score for session 3 minus session 1 for each participant. The resting state functional connectivity score for each participant was the resultant Fisher’s z transformed normalized correlation coefficient of the connectivity analysis for each voxel in the brain.

The results of the behavioral enhancement related functional connectivity analysis for sessions 1, 2, and 3 using the right precuneus as the seed region to the voxels in the entire brain are the following:

For session 1 behavioral enhancement related resting state functional connectivity was not observed for the stim–sham contrast, the stim alone contrast, or the sham alone contrast using a cluster level corrected threshold of *p* < 0.05.

**Figure 3:**
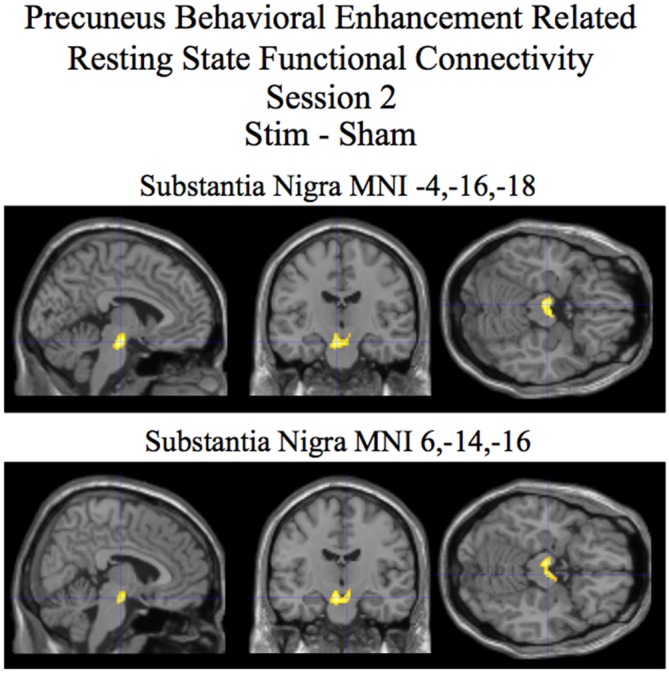
**Session 2 results of the SPM random effects between groups *t*-test for stim relative to the sham group for resting state functional connectivity with the precuneus using post-pre behavioral performance as a covariate of interest.** Statistically significant (*p* < 0.05 corrected) differences in behaviorally related resting state functional connectivity are rendered on sections of a template T1 MRI scan at MNI coordinates for the peaks in the various significant clusters. Negative “*x*” MNI coordinates denote left hemisphere and positive “*x*” values denote right hemisphere activity. For the MRI sections the right side of the image is the right side of the brain.

**Figure 4:**
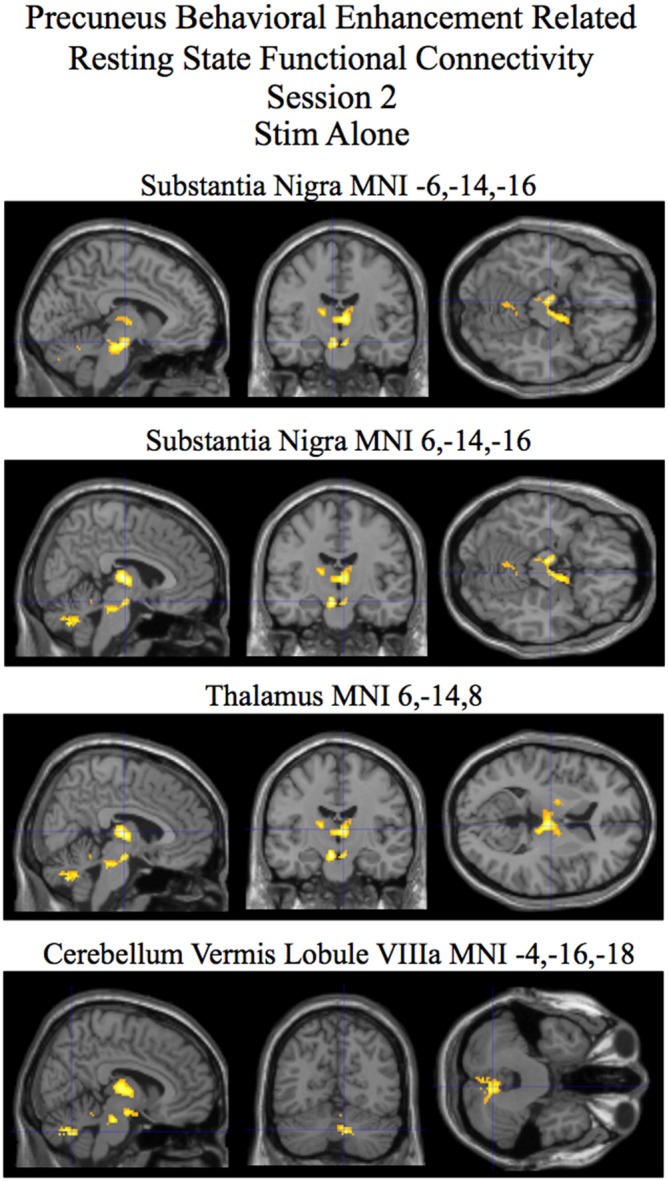
**Session 2 results of the SPM random effects *t*-test for stim alone contrast for resting state functional connectivity with the precuneus using post-pre behavioral performance as a covariate of interest.** Statistically significant (*p* < 0.05 corrected) behaviorally related resting state functional connectivity is rendered on sections of a template T1 MRI scan at MNI coordinates for the peaks in the various significant clusters. Negative “*x*” MNI coordinates denote left hemisphere and positive “*x*” values denote right hemisphere activity. For the MRI sections the right side of the image is the right side of the brain.

For session 2 a cluster encompassing the substantia nigra, red nucleus, and subthalamic nuclei was found to show statistically significant differences in behaviorally related resting state functional connectivity when correcting for multiple comparisons at the cluster level (*p* < 0.05) for the stim–sham contrast (see Figure 3 and Table 2). For the stim alone contrast three clusters showed statistically significant behavioral enhancement related resting state functional connectivity (*p* < 0.05 corrected). These clusters include the following: (1) the substantia nigra, red nucleus, and subthalamic nuclei; (2) the thalamus; and (3) the cerebellar lobule VIIIa Vermis (see Figure 4 and Table 2, bottom). The sham alone contrast for session 2 did not show any statistically significant behaviorally related resting state functional connectivity using a corrected threshold of *p* < 0.05.

**Table 2:** **Behavioral enhancement related resting state functional connectivity with the precuneus session 2**.

Brain region	MNI coordinates *x*, *y*, *z*	*T* (*df* = 24)	*Z*-score	Stim *r*	Sham *r*	Cluster size
**Stim-Sham**
Substantia Nigra	−4, −16, −18	5.20	4.22	0.86**	−0.52	200
	6, −14, −16	4.26	3.64	0.81**	−0.43	
**Stim alone**
Substantia Nigra	−6, −14, −16	6.05	4.02	0.87**		387
	6, −14, −16	4.74	3.49	0.81**		
Thalamus	6, −14, 8	5.65	3.87	0.85**		437
	−4, −12, 4	4.3	3.28	0.79**		
	−16, −14, 12	4.37	3.32	0.78**		
Cerebellum	8, −64, −34	4.91	3.57	0.82**		329
Lobule VIIIa Vermis	8, −72, −32	4.74	3.49	0.81**		
	16, −70, −36	4.70	3.47	0.81**		

Brain regions showing significant differential resting state connectivity for session 2 corrected for multiple comparisons at the cluster level (*p* < 0.05) using Monte-Carlo simulation (corrected cluster extent threshold greater than 154 contiguous voxels over uncorrected significance threshold of *p* < 0.005). BA, Brodmann area. Negative “*x*” MNI coordinates denote left hemisphere and positive “*x*” values denote right hemisphere activity. Correlation coefficient r: **denotes (*p* < 0.005).

**Figure 5:**
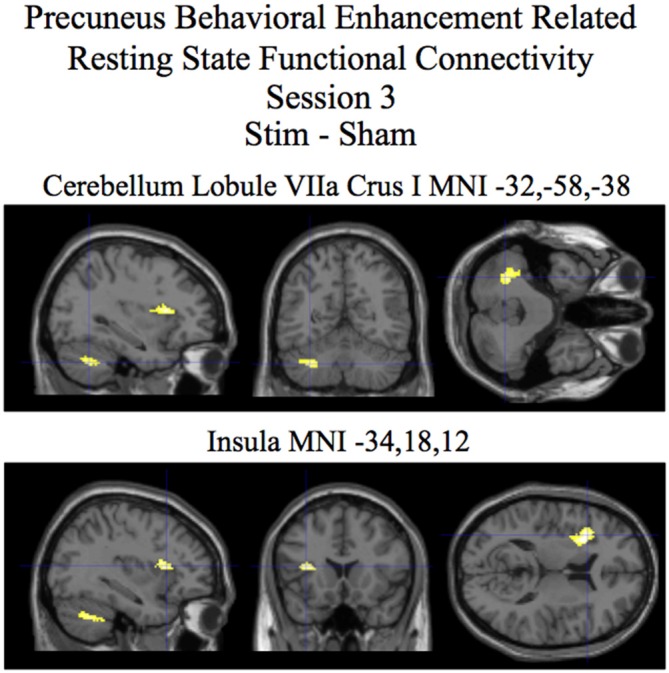
**Session 3 results of the SPM random effects between groups *t*-test for stim relative to the sham group for resting state functional connectivity with the precuneus using post-pre behavioral performance as a covariate of interest.** Statistically significant (*p* < 0.05 corrected) differences in behaviorally related resting state functional connectivity are rendered on sections of a template T1 MRI scan at MNI coordinates for the peaks in the various significant clusters. Negative “*x*” MNI coordinates denote left hemisphere and positive “*x*” values denote right hemisphere activity. For the MRI sections the right side of the image is the right side of the brain.

**Figure 6:**
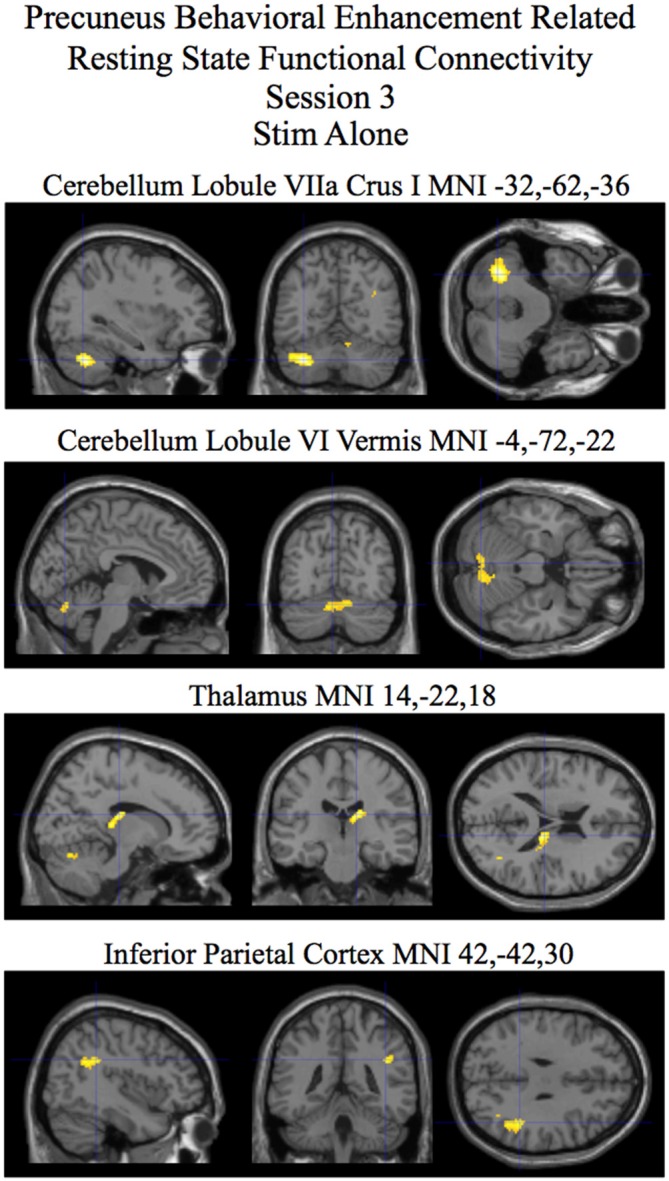
**Session 2 results of the SPM random effects *t*-test for the stim alone contrast for resting state functional connectivity with the precuneus using post-pre behavioral performance as a covariate of interest.** Statistically significant (*p* < 0.05 corrected) behaviorally related resting state functional connectivity is rendered on sections of a template T1 MRI scan at MNI coordinates for the peaks in the various significant clusters. Negative “*x*” MNI coordinates denote left hemisphere and positive “*x*” values denote right hemisphere activity. For the MRI sections the right side of the image is the right side of the brain.

For session 3 two clusters (one in the cerebellum lobule VIIa Crus I and the other in the insula) showed statistically significant (*p* < 0.05 corrected) differences in behaviorally related resting state functional connectivity for the stim–sham contrast (see Figure 5 and Table 3). For the stim alone contrast four clusters showed statistically significant (*p* < 0.05 corrected) behaviorally related resting state functional connectivity. These clusters include the following: (1) the cerebellum lobule VIIa Crus I; (2) the cerebellar lobule VI vermis and hemisphere; (3) the thalamus; and (4) the inferior parietal cortex (see Figure 6 and Table 3, bottom). The sham alone contrast for session 3 did not show any statistically significant behaviorally related resting state functional connectivity using a corrected threshold of *p* < 0.05.

**Table 3:** **Behavioral enhancement related resting state functional connectivity with the precuneus session 3**.

Brain region	MNI coordinates *x*, *y*, *z*	*T* (*df* = 24)	*Z*-Score	Stim *r*	Sham *r*	Cluster Size
**Stim–Sham**
Cerebellum Lobule VIIa Crus I	−32, −58, −38	3.54	3.15	0.82**	−0.22	174
Insula BA13	−34, 18, 12	3.77	3.31	0.67*	−0.48	186
**Stim alone**
Cerebellum Lobule VIIa Crus I	−32, −62, −36	5.52	3.82	0.85**		337
Cerebellum	−10, −76, −26	3.75	2.99	0.74**		195
Lobule VI Vermis	12, −70, −20	3.71	2.97	0.73**		
and Hemisphere	−4, −72, −22	3.64	2.93	0.72**
Thalamus	14, −22, 18	4.81	3.52	0.81**		194
	18, −32, 8	4.81	3.52	0.81**
IPC	42, −42, 30	4.46	3.36	0.79**		198

Brain regions showing significant differential resting state connectivity for session 3 corrected for multiple comparisons at the cluster level (p < 0.05) using Monte-Carlo simulation (corrected cluster extent threshold greater than 154 contiguous voxels over uncorrected significance threshold of p < 0.005). BA, Brodmann area; IPC, Inferior Parietal Cortex. Negative “x” MNI coordinates denote left hemisphere and positive “x” values denote right hemisphere activity. Correlation coefficient r: *denotes (p < 0.05) and **denotes (p < 0.005).

The magnitude and the direction of the correlation between behavioral enhancement and resting state functional connectivity are given in Tables 2, 3 (correlation coefficient *r*). For the tDCS stim group an increase in resting state functional connectivity is statistically significantly correlated (see Tables 2, 3) with the increase in behavioral performance. This relation is not found for the sham group.

## Discussion

Resting state functional connectivity during tDCS is correlated with future improvement in performance. Our study shows that tDCS affects low amplitude fluctuations in spontaneous brain activity in the precuneus region around the anodal stimulating electrode (see Figure 2, bottom and Table 1, bottom). Performance enhancement related differences (between tDCS stim and sham groups that is also present in the stim alone analysis) in resting state functional connectivity were found from the precuneus to a cluster encompassing the substantia nigra, red nucleus, and the subthalamic nuclei during concurrent tDCS stimulation for session 2 (see Figures 3, 4 and Table 2). An after-effect of tDCS stimulation on resting state functional connectivity was measured following a post-training session on the visual search task that occurred approximately 20 min after the session of tDCS stimulation. Performance enhancement related differences (between tDCS stim and sham groups that is also present in the stim alone analysis) in resting state functional connectivity were found from the precuneus to a cluster encompassing the right cerebellum lobule VIIa Crus I for session 3 (see Figures 5, 6 and Table 3).

The mechanisms behind tDCS-induced enhanced cognition have been associated with that of activity-dependent plasticity. The precuneus region revealed by the fALFF analysis to be specifically modulated by anodal tDCS is most likely the result of increased spontaneous neuronal firing due to excitability changes brought on by tDCS. Spontaneous fluctuations in BOLD signal related to cognitive abilities are known to be present at rest (Biswal et al., 1995; Stevens and Spreng, 2014). Furthermore, studies have shown that resting state activity is modulated by tDCS (Saiote et al., 2013; Clemens et al., 2014).

The results of the fALFF analysis revealed that the precuneus showed significantly greater spontaneous resting state activity for the tDCS stim over the sham group that was not attributed to preexisting differences between the groups in resting state activity present prior to tDCS stimulation (see Figure 2, bottom and Table 1, bottom). The precuneus has been found to be involved with processes related to the visual search task employed in this experiment. These include attentive tracking of moving targets (Culham et al., 1998), attention orientation (Le et al., 1998; Simon et al., 2002), attention shift between object features (Nagahama et al., 1999), and mental rotation (Suchan et al., 2002). It has been put forward that the precuneus is involved with internally guided attention and manipulation of mental images related to visuospatial processing (Cavanna and Trimble, 2006).

Using the precuneus as a seed, we were able to reveal performance related differences in resting state functional connectivity associated with tDCS stimulation. The resting state functional connectivity analysis assumes that, in the absence of ongoing task related activity, two regions that display spontaneous fluctuations in BOLD signal that are highly temporally synchronized are likely within the same functional network. Using visual search performance post-training (session 3) relative to pre-training (session 1) as a covariate of interest in this analysis allowed us to identify regions that were associated with improved performance as temporal synchrony (functional connectivity) increases with our seed ROI (precuneus). Our results revealed visual search performance enhancement related differences in resting state functional connectivity between the precuneus and a cluster encompassing primarily the substantia nigra for the stim over the sham group (that was also present for the stim group alone contrast; Figures 3, 4 and Table 2). Interestingly, consistent with the task presented in our study, previous research has implicated the substantia nigra with aspects of visuospatial processing (Matsumoto and Takada, 2013). The study by Matsumoto and Takada (2013), using single cell recordings in monkeys, showed that neurons in the substantia nigra were active when the task required visual search and working memory. Consistent with the findings in these studies, the task in our experiment required the participant to maintain the features of the target truck and distractors in working memory to accomplish the visual search task. Also relevant to our study, the substantia nigra, is part of the dopaminergic system (Björklund and Dunnett, 2007). The dopaminergic system is thought to be intimately involved with value dependent learning (Montague et al., 1996; Schultz, 1998; Doya, 2002; Callan and Schweighofer, 2008). The performance related enhancement in resting state functional connectivity for the stim over the sham group between the precuneus and the substantia nigra is consistent with the hypothesis that tDCS may in part be modulating value dependent learning systems involved with the visual search task (see Figures 3, 4 and Table 2). While we cannot rule out that the effect of tDCS stimulation alone is responsible for our observed performance related resting state connectivity, given the function of the brain regions involved, it is perhaps more likely that the effect of tDCS stimulation interacting with the visual search task is responsible for the changes in performance related resting state functional connectivity that we observe in our study.

In addition to investigating the active effects of tDCS stimulation on performance related enhancement in resting state functional connectivity we also investigated the after-effect of tDCS stimulation following a post-training session on the visual search task that occurred approximately 20 min after the session of tDCS stimulation. The session 3 results revealed visual search performance enhancement related differences in resting state functional connectivity between the precuneus and a cluster in the cerebellum lobule VIIa Crus I for the stim over the sham group (that was also present for the stim group alone contrast; Figures 5, 6 and Table 3). From studies on individuals with localized brain damage, human functional imaging studies, and animal studies the cerebellum is known to be involved with visuospatial processing (for review, see Molinari and Leggio, 2007). Related to our experimental task, the same region of the cerebellum as is present in our study has been found in an fMRI study to be involved with preparatory processes involved with visual search (Bourke et al., 2013). Also relevant is the known presence of anatomical connections between the precuneus and multiple circuits in the cerebellum through the basis pontis (Cavanna and Trimble, 2006).

One interesting finding of the analyses concerning the after-effects of tDCS stimulation is that the locus of differential performance related resting state functional connectivity (between tDCS stim and sham groups) is different from that of active tDCS stimulation. It is unclear why the focus of performance related resting state functional connectivity to the precuneus switches from the substantia nigra during active stimulation to the cerebellum as an after-effect. It is possible that the differences reflect distinctive stages of learning and correspondent modification of resting state networks.

Also of interest was the lack of any significant performance related resting state functional connectivity with the precuneus for the sham group while several regions were found to be significant for the tDCS stim group for active and after-effect analyses (see Figures 4, 6 and Tables 2, 3). One possibility is that multiple degenerate networks (Edelman, 1987) are utilized for processing the visual search task for the sham group whereas for the tDCS stim group, as a result of stimulation, specific networks are selectively used. This is potentially why the degree of correlation between the strength of these networks and behavioral improvement in performance is relatively high (see Tables 2, 3) for the tDCS stim group.

In our study as well as in others (e.g., Polanéa et al., 2011) the seed ROIs for the resting state functional connectivity analyses were selected based on previous, although different, analyses of the same data. The advantage of using the results of the fALFF analysis as seed ROIs is that we ensure that we are actually utilizing regions that are showing potential modulation as a result of the tDCS for the functional connectivity analysis instead of arbitrarily selecting a region underneath the stimulation electrode. We do not believe that this will unduly bias the results of the functional connectivity analyses for stim over the sham group comparison because the fALFF (fluctuations in low frequency activity in single voxels) and functional connectivity (correlation in time course between voxels) analyses are quite different. Additionally, we employed the use of improvement in behavioral performance post-relative to pre-training as a covariate of interest in the functional connectivity analyses. There is no* a priori* reason to believe that future improvement in behavioral performance should be predicted by differences in fALFF or functional connectivity unless of course these changes are induced as a result of tDCS.

As with all brain imaging studies, there are many potential limitations and confounds that need to be addressed. One potential limitation of this study is that the changes we see in fALFF and resting state functional connectivity may not be a result of changes in spontaneous neural activity, but rather may be a result of changes in cerebral perfusion or noise induced by tDCS (this is only a potential problem for session two in which concurrent tDCS and fMRI was applied). Previous studies using concurrent tDCS and fMRI have suggested that tDCS induced distortions on fMRI SNR are minimal (Antal et al., 2011; Zheng et al., 2011). It is unlikely that these tDCS distortion effects would be specific to the low frequency range (0.01–0.08 Hz) used in the fALFF analysis. Since the fALFF compares this low frequency range to the entire range (0–0.25 Hz) it would cancel out any effects induced by tDCS (artifacts on fMRI, etc …) that are not frequency specific. In terms of the resting state functional connectivity analyses we employed there is no reason why changes in cerebral perfusion or noise induced by tDCS would correlate with post-relative to pre-training behavioral performance. It is much more likely that the behaviorally related resting state functional connectivity we observed is a result of changes in spontaneous neural activity resulting from tDCS stimulation.

An additional confound that we did not test was whether the radio frequency and gradients associated with fMRI EPI scanning influences the tDCS current. The NeuroConn DC-Stimulator MR that we used for this experiment includes an RF filter module with MRI compatible cables and electrodes. These components help to prevent effects of RF on tDCS current. In addition we avoided cable loop formation in the setup that may result in gradient induced currents. Furthermore, the fMRI compatible cables used for tDCS had a high resistance (5 kΩ), which should also decrease the induced current avoiding potential effects of MRI on tDCS. Although we do not believe it to be the case, since we did not measure the current during stimulation, we cannot rule out the possibility that the tDCS current could have been reduced or modulated such that its enhancement effects on behavior were diminished.

One of the biggest limitations of our study was the lack of a robust difference in behavioral performance after training for the tDCS stim over the sham group. There are several reasons why group differences in improved performance between the stim and sham groups may not have been observed in our study when they have been found in many previous studies (Coffman et al., 2014). One reason may be that the task was too difficult when first starting such that many participants were at near chance levels. The effects of training were so great in this situation that the modulatory benefits of tDCS were washed out in the behavioral data. Another reason for a lack of a behavioral performance enhancement difference between tDCS stim and sham groups may be that training was too short for the modulatory effects of tDCS to be revealed in behavioral performance. Relatedly, another limitation in our study was the restriction on the level of tDCS stimulation to be a maximum of 1 mA with our version of the NeuroConn DC-Stimulator MR. Many tDCS studies commonly utilize 2 mA to get robust enhancement in behavioral performance (Coffman et al., 2014). One reason why we did not see an overall difference between tDCS stim and sham groups may be because the level of stimulation was too low to induce robust enhancement in the short training time of approximately 16 min. Given that we do see strong performance related differences in the resting state functional connectivity data it may be the case that we are observing the early stages of tDCS modulated learning. The involvement of dopaminergic brain regions involved with value dependent learning as well as to working memory and visual attention are certainly consistent with this hypothesis. In the future it would be interesting to investigate whether longer training on this same task as well as higher stimulating levels (2 mA) results in enhanced behavioral performance for tDCS stim over sham groups.

## Conclusion

Participants’ who showed greater resting state functional connectivity between parietal regions and dopaminergic subcortical brain regions (substantia nigra, hippocampus, and amygdala) showed greater improvement in visual search task performance. Essentially, future improvement in performance showed a significant linear relationship with resting state functional connectivity (thought to be) induced by tDCS. These results suggest that it may be possible to employ multivariate pattern analysis machine learning decoding techniques to predict future performance, given a certain pattern of resting state functional connectivity. Future experiments need to be conducted to determine if changes in resting state functional activity and connectivity induced by tDCS can be used to predict long-term changes in task performance. One could even use neurofeedback techniques (Fukuda et al., 2015) in conjunction with tDCS to induce greater changes in specific functional networks. These techniques could be utilized to optimize performance benefits resulting from tDCS. Our results have wide ranging implications regarding effective utilization of tDCS for neuroergonomic as well as therapeutic, and rehabilitative applications.

## Dedication

The present manuscript wishes to be the authors’ personal honor to the memory of Raja Parasuraman, as a tribute to his scientific competence, and the contribution that he gave to our research projects in these years of proficient collaboration. Raja was a kind and generous man who was a pioneer in the field of neuroergonomics. He passionately sought out to facilitate and inspire researchers in the field instilling a thirst for knowledge. RP will always be present as a driving force inspiring us all to succeed.

## Author Contributions

DEC, BF and AW conducted experiment. DEC, BF, AW and RP designed experiment, analyzed the data and wrote the manuscript.

## Funding

This work was supported by the Center for Information and Neural Networks, National Institute of Information and Communications Technology. Additional support to BF was given by the National Science Foundation sponsored East Asia and Pacific Summer Institutes fellowship award 1414852, funded in collaboration with Japan Society for the Promotion of Science. Additional support to RP was given by the Air Force Office of Sponsored Research grant FA9550-10-1-0385.

## Conflict of Interest Statement

The authors declare that the research was conducted in the absence of any commercial or financial relationships that could be construed as a potential conflict of interest.
